# Trevo 6 × 25mm vs. 4 × 30mm in Mechanical Thrombectomy of M1 LVO

**DOI:** 10.3389/fneur.2021.677630

**Published:** 2021-09-29

**Authors:** Marion John Oliver, Emily Brereton, Muhib A. Khan, Alan Davis, Justin Singer

**Affiliations:** ^1^Department of Neurology, University of Toledo, Toledo, OH, United States; ^2^College of Human Medicine, Michigan State University, East Lansing, MI, United States; ^3^Spectrum Health, Grand Rapids, MI, United States

**Keywords:** stent retreiver, large vessel occlusion, neurointervention, mechanical thrombectomy, stroke

## Abstract

**Objectives:** Our primary objective was to determine the successful rate of recanalization of M1 large vessel occlusion using either the Trevo 4 × 30 mm or 6 × 25 mm stent during mechanical thrombectomy. Our secondary objectives were to determine differences between the use of these two stent retrievers regarding first-pass effect, periprocedural complications, and mortality in the first 90 days.

**Methods:** This is a retrospective cohort study. Data regarding the stent used, recanalization, number of passes, periprocedural complications, and mortality were determined *via* our mechanical thrombectomy database along with chart review.

**Conclusion:** When comparing Trevo 4 × 30 mm to 6 × 25 mm stent retrievers used in mechanical thrombectomy for middle cerebral artery large-vessel occlusion causing stroke, there is no statistically significant difference in successful recanalization rates, first-pass effect, perioperative complications, or mortality at 90 days. Studies like this will hopefully lead to further prospective, randomized controlled trials that will help show experts in the field an additional way to perform this procedure effectively and safely.

## Introduction

Acute ischemic stroke secondary to large-vessel occlusion accounts for a significant amount of morbidity and mortality in the world (1). In the USA, stroke is the fifth leading cause of death (2). Of the people that die from stroke, an overwhelming proportion of them are due to large-vessel occlusion (LVO) (3, 4).

Recent clinical trials have shown improved functional outcomes of mechanical thrombectomy in patients with LVO (5–9). The success of these recent trials as compared to earlier studies is related to the evolution of mechanical thrombectomy technology along with improved patient selection. Specifically, trials like SWIFT and TREVO II show the importance of stent retrievers like Solitaire and Trevo compared to Merci catheters (10).

These stent retrievers differ in various ways, including but not limited to size, shape, and material. There is paucity of data on which Trevo stent retriever is better for achieving recanalization efficiently. Our study looked to answer the question of which Trevo stent retriever is superior, Trevo 4 × 30 mm or 6 × 25 mm, in recanalization of M1 LVO.

## Methods

### Design

This was a retrospective cohort study based out of a single comprehensive stroke center setting. Retrospective chart and mechanical thrombectomy database review was performed over a 2-year period between 2018 and 2019. IRB approval was obtained, and due to the retrospective nature of the study, informed consent was not necessary.

### Patient Selection

There were 86 patients included in this retrospective study from 2018 to 2019. Inclusion criteria were the following: patients >18 years of age, have had a mechanical thrombectomy between the time from 2018 to 2019, have LVO in the M1 segment confirmed on CTA and CTP causing acute ischemic stroke, ≤ 24 h from the last known well, only mechanical thrombectomy done with Trevo 4 × 30 mm or 6 × 25 mm stent retrievers, and all or a portion of subjects care at a primary stroke center.

### Data Collection

Patients were divided into two main groups based on which stent retriever was used, and their revascularization was recorded based on their TICI score (11). The TICI score was recorded by the interventionalist that performed the procedure. Additional information that was collected included the following: number of passes, initial NIHSS, discharge NIHSS, change in NIHSS, patient's age, tobacco use, atrial fibrillation, diabetes, hypertension, hyperlipidemia, re-occlusion, groin hematoma, vasospasm, dissection, intracerebral hemorrhage, subarachnoid hemorrhage, use of balloon guided catheterization (BGC), tPA administration, specific vessel occlusion, and 90-day mortality (see Table 1).

**Table 1:** Demographic and clinical characteristics.

	**4 x 30 mm stent retriever**	**6 x 25 mm stent retriever**	***p*-value**
Age^*^	69.1+18.3	70.4+18.7	0.745
Sex (% female)	30/50 (60.0%)	12/36 (33.3%)	0.015
Hypertension	37/50 (74.0%)	28/36 (77.8%)	0.687
HLD	25/50 (50.0%)	23/36 (63.9%)	0.201
Diabetes	7/50 (14.0%)	8/36 (22.2%)	0.322
Afib	19/50 (38.0%)	13/36 (36.1%)	0.858
Tobacco use			0.881
Former	9/50 (18.0%)	8/36 (22.2%)	
Current	14/50 (28.0%)	10/36 (27.8%)	
Never	27/50 (54.0%)	18/36 (50.0%)	
Tpa	21/50 (42.0%)	11/36 (30.6%)	0.367
Vessel			0.799
LMCA	25/50 (50.0%)	19/36 (52.8%)	
RMCA	25/50 (50.0%)	17/36 (47.2%)	
BGC	34/50 (68.0%)	2/36 (5.60%)	<0.001
Puncture to perfusion time^†^	20 (12, 28)	20 (11, 41)	0.996
Initial NIHSS^†^	15 (10, 22)	19 (11.5, 24.5)	0.146
Passes^†^	1 (1, 2)	1 (1, 2)	0.888

*HLD, Hyperlipidemia; Afib, Atrial fibrillation; tPA, Tissue plasminogen activator; LMCA, Left main coronary artery; RMCA, Right main coronary artery; BGC, Balloon guided catheterization; NIHSS, National Institutes of Health Stroke Scale*.

* *mean + SD*.

† *median (25^th^ percentile, 75^th^ percentile)*.

### Primary Outcome Variable

The primary outcome variable was successful recanalization, as defined by thrombolysis in cerebral infarction (TICI) score of IIb–III.

### Secondary Outcome Variables

Secondary outcome variables are first-pass effect, perioperative complications, and mortality at 90 days.

### Statistical Methods

Summary statistics were calculated. Age is expressed as the mean ± SD, while all other quantitative data are expressed as the median, with the interquartile range in parentheses (25th percentile, 75th percentile). Nominal data are expressed as a percentage. Comparison between groups for age was performed using the *t*-test, while comparisons for all other quantitative variables were performed using the Mann–Whitney U test. Nominal variables were evaluated using the chi-square test or the Fisher's exact test, as appropriate. Significance was assessed at p <0.05. Analyses were performed using Stata v. 15.1 (StataCorp, College Station, TX, USA).

## Results

- Eighty-six patients were included in this retrospective study from 2018 to 2019.
- Trevo 4 × 30 mm and 6 × 25 mm had successful recanalization 96.0 and 91.7%, respectively, *p* = 0.645.
- Ninety-day mortality was 16.0% for 4 × 30 mm and 27.8% for 6 × 25 mm, *p* = 0.185.
- tPA was used in 42% of the 4 × 30 mm group and 30.6% of the 6 × 25 mm group.
- Vessels occluded were 50.0% LMCA for 4 × 30 mm, 52.8% 6 × 25mm, and RMCA 50.0% 4 × 30 mm group, 47.2% 6 × 25 mm group.
- BGC was used 68% of the 4 × 30 mm and 5.6% of the 6 × 25 mm groups, *p* < 0.001.
- SAH was a complication in 4% in the 4 × 30 mm group and 2.80% in the 6 × 25 mm, *p* > 0.999.
- ICH was a complication in 2% in the 4 × 30 mm group and 0% in the 6 × 25 mm group, *p* > 0.999.
- Groin hematoma was a complication 0% of the 4 × 30 mm group and 2.8% of the 6 × 25 mm group, *p* = 0.419.
- Vessel perforation took place in 2% of the 4 × 30 mm group and 0% of the 6 × 25 mm group, *p* > 0.999.
- Arterial dissection, vasospasm, and re-occlusion were not complications in either group (see Table 2).

**Table 2:** Outcomes.

	**4 x 30 mm stent retriever**	**6 x 25 mm stent retriever**	***p*-value**
90 day mortality	8/50 (16.0%)	10/36 (27.8%)	0.185
Successful recanalization^*^	48/50 (96.0%)	33/36 (91.7%)	0.645
SAH	2/50 (4.00%)	1/36 (2.80%)	>0.999
ICH	1/50 (2.00%)	0/36 (0.00%)	>0.999
Dissection	0/50 (0.00%)	0/36 (0.00%)	>0.999
Vasospasm	0/50 (0.00%)	0/36 (0.00%)	>0.999
Groin hematoma	0/50 (0.00%)	1/36 (2.80%)	0.419
Re-occlusion	0/50 (0.00%)	0/36 (0.00%)	>0.999
Vessel perforation	1/50 (2.00%)	0/36 (0.00%)	>0.999
Discharge NIHSS^†^	6 (1, 11.5)	5 (2, 14)	0.550
Delta NIHSS^†^^,^ ^‡^	8 (3, 14)	13 (2, 18)	0.135

*SAH, Subarachnoid hemorrhage; ICH, Intracerebral hemorrhage; TICI, Thrombolysis in cerebral infarction; NIHSS, National Institutes of Health Stroke Scale*.

* * TICI of 2b or 3*.

† * median (25^th^ percentile, 75^th^ percentile)*.

‡ * Initial NIHSS – Discharge NIHSS*.

## Discussion

There are many combinations of techniques that are used in mechanical thrombectomy. *In this study, we included two operators that used continuous aspiration prior to intracranial vascular embolectomy (CAPTIVE) and stent retriever-assisted vacuum-locked extraction (SAVE) techniques. One preferred the Trevo 6* × *20 mm stent without the use of BGC and the other Trevo 4* × *30 mm stent with the use of BGC. All other equipment was identical*.

Limited data is available for guidance as to which stent retriever to use for M1 LVO mechanical thrombectomy. Interventionalists make stent size decisions based on how they were trained, which vessel is affected, their own personal experiences, and/or the measurement of the vessel itself on imaging. We had two operators that used both.

This study initially proposed that the larger stent diameter would achieve greater revascularization compared to the smaller stent diameter. This hypothesis was based on the larger stent diameter having greater radial force in the artery of interest.

One study by Machi et al. evaluates properties of various stent retrievers as well as their effectiveness. One specific parameter measured was radial force, both outward and inward. Outward radial force was greater in the larger-diameter stent retriever compared to the smaller-diameter stent retriever. Inward radial force varied considerably based on the diameter of the tube it was tested in (12). Additional Trevo data along with one retrospective study by Yi et al. state that larger stent diameter has larger radial force regardless of the vessel size, which in turn leads to better angiographic and clinical outcomes (13, 14).

Another point to consider is the length of the stent. Expert opinion in the field states that the longer the length of the stent, the greater the odds of proper clot integration, thus yielding a greater revascularization rate. This concept leaves radial force behind. The STRATIS registry publication by Zaidat et al. showed that increased length of Solitaire stent retrievers demonstrates a higher rate of first-pass effect and modified first-pass effect compared to larger-diameter or shorter-stent retrievers. However, final revascularization or significant differences in functional dependence/mortality at 90 days post procedure were not seen (15). Our results show us that neither the diameter nor the length of the Trevo stent retriever has a statistically significant effect on successful revascularization, first-pass effect, perioperative complications, or mortality at 90 days. *Mortality of 27.8% in the Trevo 6* × *20 mm group compared to 16.0% was not statistically significant; however, we do believe it is important to be noted*.

The use of balloon-guided catheterization (BGC) was statistically significant in the 4 × 30 group compared to the 6 × 25 group. Despite the statistical significance of the use of BGC in 4 × 30 group, there was no statistical significance achieved in terms of primary endpoint compared to 6 × 25 without the use of BGC. Several studies have shown that regardless of thrombectomy techniques used by the interventionalist (ADAPT, SAVE, CAPTIVE, etc.), the use of BGC is an independent predictor of higher rate of successful revascularization, first-pass effect, and mRS 0–2 at 90 days. The advantages of BCG are flow arrest proximal to the clot and the decreasing arterial pressure that impacts the clot. This leads to a reduction in distal embolization along with enhancement of the actual thrombectomy technique (16).

Our study does have its limitations. It was retrospective, had a limited sample size due to our strict exclusion criteria, and was not a randomized control trial. Additionally, the use of BGC and a lack of control in length or width of stent size were limitations as well. Perhaps our primary endpoint results would have been different if the 4 × 30 group had not used BCG or the 6 × 25 group had used BGC.

Despite these limitations, this study challenges us to think about which technique/tools for this procedure are best. Additional randomized trials are needed to further best clarify the optimal approach for angiographic outcomes, first pass, perioperative outcomes, and 90-day mortality.

## Data Availability Statement

The raw data supporting the conclusions of this article will be made available by the authors, without undue reservation.

## Ethics Statement

Ethical review and approval was not required for the study on human participants in accordance with the local legislation and institutional requirements. Written informed consent for participation was not required for this study in accordance with the national legislation and the institutional requirements.

## Author Contributions

Lead author MO. Principle investigator was JS. All authors contributed to the article and approved the submitted version.

## Conflict of Interest

JS is a consultant for Stryker and Medtronic. The remaining authors declare that the research was conducted in the absence of any commercial or financial relationships that could be construed as a potential conflict of interest.

## Publisher's Note

All claims expressed in this article are solely those of the authors and do not necessarily represent those of their affiliated organizations, or those of the publisher, the editors and the reviewers. Any product that may be evaluated in this article, or claim that may be made by its manufacturer, is not guaranteed or endorsed by the publisher.
