# Supplementary material for: Trevo 6 × 25mm vs. 4 × 30mm in Mechanical Thrombectomy of M1 LVO
Source: Front Neurol. 2021 Sep 29;12:677630. doi: 10.3389/fneur.2021.677630 (PMC8511397; doi:10.3389/fneur.2021.677630)
Supplement: Supplementary file 1 [file Data_Sheet_1.PDF]

Table 2. Outcomes

	4x30 mm stent retriever	6x25 mm stent retriever	p-value
90 day mortality	8/50 (16.0%)	10/36 (27.8%)	0.185
Successful recanalization*	48/50 (96.0%)	33/36 (91.7%)	0.645
SAH	2/50 (4.00%)	1/36 (2.80%)	> 0.999
ICH	1/50 (2.00%)	0/36 (0.00%)	> 0.999
Dissection	0/50 (0.00%)	0/36 (0.00%)	> 0.999
Vasospasm	0/50 (0.00%)	0/36 (0.00%)	> 0.999
Groin hematoma	0/50 (0.00%)	1/36 (2.80%)	0.419
Re-occlusion	0/50 (0.00%)	0/36 (0.00%)	> 0.999
Vessel perforation	1/50 (2.00%)	0/36 (0.00%)	> 0.999
Discharge NIHSS†	6 (1, 11.5)	5 (2, 14)	0.550
Delta NIHSS†, ‡	8 (3, 14)	13 (2, 18)	0.135

SAH: Subarachnoid hemorrhage; ICH: Intracerebral hemorrhage; TICI: Thrombolysis in cerebral infarction; NIHSS: National Institutes of Health Stroke Scale

* TICI of 2b or 3

† median (25th percentile, 75th percentile)

‡ Initial NIHSS – Discharge NIHSS
