# Supplementary material for: Trevo 6 × 25mm vs. 4 × 30mm in Mechanical Thrombectomy of M1 LVO
Source: Front Neurol. 2021 Sep 29;12:677630. doi: 10.3389/fneur.2021.677630 (PMC8511397; doi:10.3389/fneur.2021.677630)
Supplement: Supplementary file 2 [file Data_Sheet_2.PDF]

Table 1. Demographic and clinical characteristics

| | 4x30 mm stent retriever | 6x25 mm stent retriever | p-value |
|---|---|---|---|
| Age* | 69.1±18.3 | 70.4±18.7 | 0.745 |
| Sex (% female) | 30/50 (60.0%) | 12/36 (33.3%) | 0.015 |
| Hypertension | 37/50 (74.0%) | 28/36 (77.8%) | 0.687 |
| HLD | 25/50 (50.0%) | 23/36 (63.9%) | 0.201 |
| Diabetes | 7/50 (14.0%) | 8/36 (22.2%) | 0.322 |
| Afib | 19/50 (38.0%) | 13/36 (36.1%) | 0.858 |
| Tobacco use | | | 0.881 |
| Former | 9/50 (18.0%) | 8/36 (22.2%) | |
| Current | 14/50 (28.0%) | 10/36 (27.8%) | |
| Never | 27/50 (54.0%) | 18/36 (50.0%) | |
| tPA | 21/50 (42.0%) | 11/36 (30.6%) | 0.367 |
| Vessel | | | 0.799 |
| LMCA | 25/50 (50.0%) | 19/36 (52.8%) | |
| RMCA | 25/50 (50.0%) | 17/36 (47.2%) | |
| BGC | 34/50 (68.0%) | 2/36 (5.60%) | < 0.001 |
| Puncture to perfusion time† | 20 (12, 28) | 20 (11, 41) | 0.996 |
| Initial NIHSS† | 15 (10, 22) | 19 (11.5, 24.5) | 0.146 |
| Passes† | 1 (1, 2) | 1 (1, 2) | 0.888 |

HLD: Hyperlipidemia; Afib: Atrial fibrillation; tPA: Tissue plasminogen activator; LMCA: Left main coronary artery; RMCA: Right main coronary artery; BGC: Balloon guided catheterization; NIHSS: National Institutes of Health Stroke Scale

* mean±SD

† median (25th percentile, 75th percentile)
